# The Geographic Distribution of a Tropical Montane Bird Is Limited by a Tree: Acorn Woodpeckers (*Melanerpes formicivorus*) and Colombian Oaks (*Quercus humboldtii*) in the Northern Andes

**DOI:** 10.1371/journal.pone.0128675

**Published:** 2015-06-17

**Authors:** Benjamin G. Freeman, Nicholas A. Mason

**Affiliations:** 1 Department of Ecology and Evolutionary Biology, Cornell University, Ithaca, New York, United States of America; 2 Cornell Lab of Ornithology, Ithaca, New York, United States of America; Aristotle University of Thessaloniki, GREECE

## Abstract

Species distributions are limited by a complex array of abiotic and biotic factors. In general, abiotic (climatic) factors are thought to explain species’ broad geographic distributions, while biotic factors regulate species’ abundance patterns at local scales. We used species distribution models to test the hypothesis that a biotic interaction with a tree, the Colombian oak (*Quercus humboldtii*), limits the broad-scale distribution of the Acorn Woodpecker (*Melanerpes formicivorus*) in the Northern Andes of South America. North American populations of Acorn Woodpeckers consume acorns from *Quercus* oaks and are limited by the presence of *Quercus* oaks. However, Acorn Woodpeckers in the Northern Andes seldom consume Colombian oak acorns (though may regularly drink sap from oak trees) and have been observed at sites without Colombian oaks, the sole species of *Quercus* found in South America. We found that climate-only models overpredicted Acorn Woodpecker distribution, suggesting that suitable abiotic conditions (e.g. in northern Ecuador) exist beyond the woodpecker’s southern range margin. In contrast, models that incorporate Colombian oak presence outperformed climate-only models and more accurately predicted the location of the Acorn Woodpecker’s southern range margin in southern Colombia. These findings support the hypothesis that a biotic interaction with Colombian oaks sets Acorn Woodpecker’s broad-scale geographic limit in South America, probably because Acorn Woodpeckers rely on Colombian oaks as a food resource (possibly for the oak’s sap rather than for acorns). Although empirical examples of particular plants limiting tropical birds’ distributions are scarce, we predict that similar biotic interactions may play an important role in structuring the geographic distributions of many species of tropical montane birds with specialized foraging behavior.

## Introduction

Understanding the factors that explain species’ distributional limits is a fundamental goal of ecology and biogeography [1]. Abiotic factors such as temperature and precipitation are strong predictors of species richness [2], and several disparate lines of evidence support the hypothesis that abiotic factors often set species’ broad geographic distributions (the geographic Grinnelian niche) [3]—meta-analyses reveal that species’ range limits are often determined by abiotic conditions [4,5], many species are shifting their distributions to cooler upslope or higher-latitude environments in concordance with modern global warming [6,7], and introduced species’ distributional limits can often be predicted from the climatic conditions they experience in their native range [8,9].

However, climate is not the only factor that influences species’ distributional limits. Biotic factors such as habitat variables, resource availability and species interactions (i.e., competition, mutualism and predation) can all limit distributions [1,10]. Many examples demonstrate that species interactions can influence distributional limits at fine spatial scales (e.g., for competition) [11–15], and the influence of biotic factors such as interspecific competition can also explain non-random abundance patterns at regional scales [16,17]. Thus, it is clear that biotic factors can impact patterns of species’ abundance and distribution on small spatial scales (the local Eltonian niche) [3]. However, the relative paucity of examples where biotic factors explain species’ geographic limits supports the Eltonian noise hypothesis, which posits that biotic interactions seldom influence species’ geographic extents [18]. Investigating the influence of biotic interactions on species’ distributions is an active arena of research [10,19–21], and an increasing number of case studies demonstrate that biotic interactions can be important factors influencing species’ distributions [22–25].

**Fig 1:**
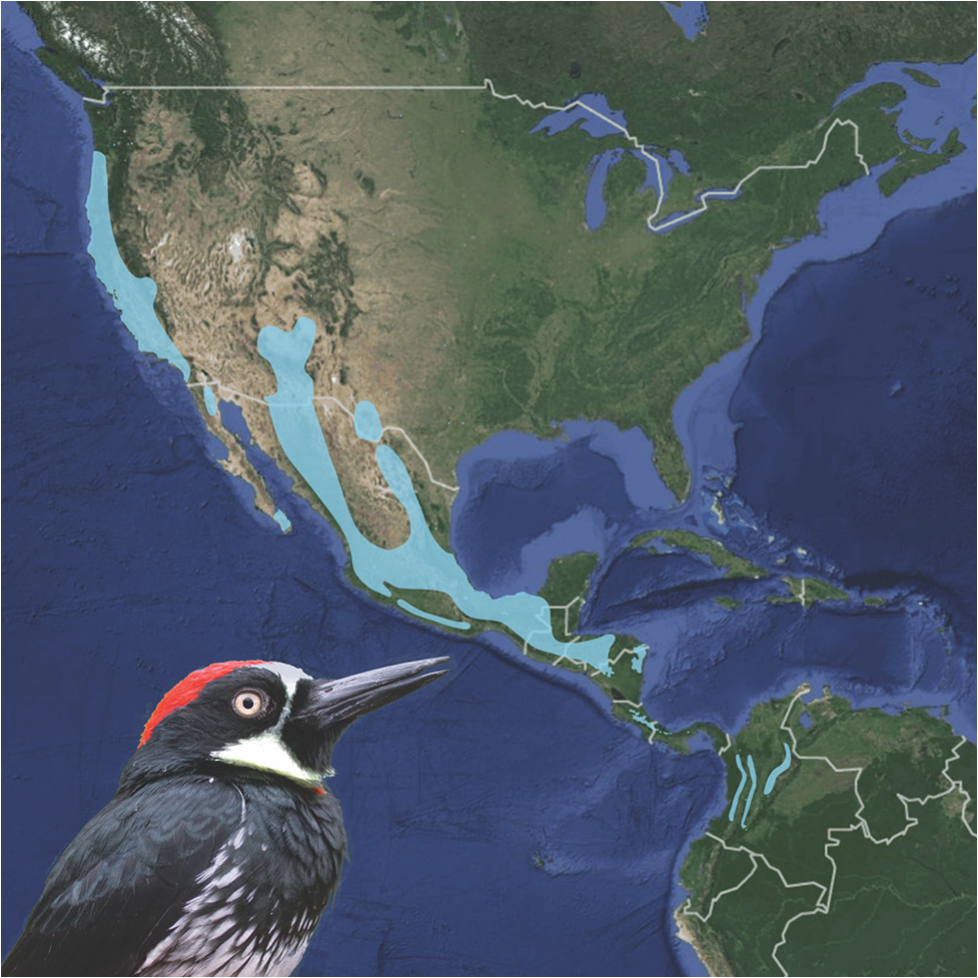
Geographic distribution of Acorn Woodpecker (*Melanerpes formicivorus*). An expert range map of Acorn Woodpecker from BirdLife International is shown in light blue. Inset shows a male Acorn Woodpecker (photograph by Walt Koenig). Reprinted under a CC BY license, with permission from Walt Koenig, original copyright 2011.

We provide one of the first tests comparing the relative influence of climate and a putatively strong biotic interaction in limiting the geographic distribution of the tropical population of a widespread bird species found in both temperate and tropical biomes in the Americas. We used a species distribution modeling approach to investigate two non mutually exclusive factors that could limit the distribution of the Acorn Woodpecker (*Melanerpes formicivorus*) at its southern range margin: 1) abiotic factors such as temperature, precipitation and seasonality, or 2) a biotic interaction with a putatively important food resource. As its name implies, acorns produced by *Quercus* oaks form an important component of the Acorn Woodpeckers’ diet, at least within North America, where woodpeckers store acorns in granary trees [26]. These stored acorns are then eaten by woodpeckers during periods of low resource availability (e.g., winter; [26]). This woodpecker-oak interaction is sufficiently strong that Acorn Woodpecker distribution along the Pacific coast of North America is effectively limited to locations where multiple species of oaks co-occur [27]. However, Acorn Woodpeckers inhabit a wide latitudinal distribution from western North America south to the Northern Andes in South America, and the influence of oak distributions on Acorn Woodpecker distribution in other regions remains unknown (Fig 1).

Biogeographic and ecological viewpoints provide conflicting perspectives to the hypothesis that oak presence limits the Acorn Woodpecker’s southern range margin. On one hand, the Acorn Woodpeckers’ southern range margin roughly correlates with the distribution of the sole *Quercus* species in South America, the Colombian oak (*Quercus humboldtii*) [28], but does not correspond with an obvious biogeographical or climatic barrier, suggesting that the woodpecker’s distribution may be limited more by the occurrence of oaks than by climatic conditions alone. Indeed, the Acorn Woodpecker’s distributional limits are unusual among Andean bird species: montane bird species that co-occur with Acorn Woodpeckers in southern Colombia typically range farther south into neighboring Ecuador [29]. On the other hand, Acorn Woodpeckers in Colombia can be found at sites without Colombian oaks, have a varied diet including insects and fruit, and do not appear to rely on acorns as a food resource (but do regularly drink sap from Colombian oak trees) [30]. Thus, documented ecological interactions between Colombian oaks and Acorn Woodpeckers within Colombia are unclear and suggest the possibility that Colombian oaks may not influence the woodpeckers’ distribution.

We used a species distribution modeling approach to test the hypothesis that Acorn Woodpecker distribution at its southern range margin is limited by a biotic interaction with Colombian oaks. To test this hypothesis, we compared the performance of three species distribution models. The first model (“abiotic” model) was a standard climate envelope model built using Acorn Woodpecker occurrence data and the climatic variables associated with woodpecker presence localities. The second model (“Quercus” model) included a single, binary layer that reflects the presence or absence of *Quercus humboldtii* based on occurrence records. The third model (“abiotic and Quercus” model) included both Colombian oak presence-absence as a biotic layer and climatic variables. The hypothesis that an interaction with Colombian oaks limits Acorn Woodpecker distribution in the Northern Andes makes the general prediction that models including the biotic layer (both the “Quercus” and the “abiotic and Quercus” models) will outperform the model without the biotic layer (the “abiotic” model). We assessed this prediction by comparing the performance of the three species distribution models using several metrics. The hypothesis further predicts that models that include biotic layers will more accurately model the Acorn Woodpeckers’ southern range limit than the abiotic model. We compared model overprediction (false positives, quantified as the commission rate) beyond the Acorn Woodpecker’s southern range limit for the three models to test this specific prediction.

## Materials and Methods

### Spatial Data

We used occurrence data downloaded from the Global Biodiversity Information Facility (GBIF; http://www.gbif.org/) to develop a database of georeferenced Acorn Woodpecker localities in the Northern Andes of Colombia (hereafter ‘Northern Andes’). A large proportion of occurrence data came from citizen science observational data entered into eBird [31]. To minimize georeferencing errors from observational data, we accepted eBird records only if they came from stationary counts (checklists of bird species detected by a stationary observer from a single geographic point), exhaustive area counts with a total area < 1 km^2^ (checklists of bird species detected by an observer within a region < 1 km^2^), or traveling counts that covered < 5 km (checklists of bird species detected by an observer traveling < 5 km). We included 319 Acorn Woodpecker localities from South America in our finalized data set (S1 File). To avoid issues associated with geographic sampling bias, we used the spThin package [32] to subsample our dataset such that all occurrence records were separated by a minimum distance of 1 km. We retained 113 occurrence records after thinning. This number of occurrence records is sufficient to generate robust species distribution models; reliable habitat suitability models have been built with far fewer occurrence points (i.e., < 25) in other empirical studies [33,34].

Colombian oaks are the southernmost species of *Quercus* oak in the Americas, and the only species found in South America. Endemic to the Western, Central and Eastern Andes of Colombia, Colombian oaks live in montane forests (1,500–3,300 m) where they sometimes grow in nearly monospecific stands termed ‘robledales’ [28]. We used a database of 117 georeferenced Colombian oak records from Gonzalez et al. [35] in our analysis (S2 File). This database includes records from herbaria and field surveys, and was quality checked by experts [35].

### Species distribution modeling

We randomly partitioned one quarter (28 records) of the Acorn Woodpecker occurrence records to test the performance of species distribution models built with the remaining data (85 records), a methodology known as *k*-fold partitioning. We downloaded data for 19 abiotic variables at a resolution of 30 arc seconds from the WorldClim database [36]. Because our study focuses on Acorn Woodpeckers in the Northern Andes, we limited our species distribution modeling to an extent between 69°W and 82°W longitude and 7°S and 11°N latitude, which does not include the nearest neighboring population of Acorn Woodpeckers present in the highlands of Costa Rica and Panama. We used 250 randomly generated “pseudo-absence” points within this geographic extent for model training, and constructed species distribution models using Maxent v3.3.3k [37,38] with default settings using the ‘dismo’ package [39].

Model complexity, or the number of variables included in a model, can affect performance and error rates when predicting species distributions. Maxent includes a built-in level of protection against overfitting models, known as L_1_ regularization. This procedure uses a parameter (*β*) that weights the penalty applied to the addition of extra parameters and is automatically adjusted by recent versions of Maxent [37]. Recently, Warren and Seifer [40] demonstrated that overparameterized Maxent models consistently performed better than underparameterized Maxent models. Thus, we opted to let Maxent control model parameterization and included all 19 bioclim variables as input into Maxent, as done in other empirical studies [41–43].

We also generated a grid of Colombian oak presence or pseudo-absence as a binary character by extending known Colombian oak localities to include a radius of 20 km. We used the same set of training and testing data to compare the performance between species distribution models constructed from solely abiotic variables, a single biotic layer (presence-absence of *Quercus*), and one that included Colombian oak presence or absence in addition to the same abiotic variables.

We assessed the contribution of each layer, such as bioclim variables or the presence of *Quercus* oaks, towards generating the final model in Maxent. Maxent estimates the importance of each variable in two ways, first by estimating a heuristic approach to calculate the scaled ‘percent contribution’ of each variable, which reflect increases in model performance. However, this number can be influenced by collinear relationships with other layers and must be interpreted with caution. To address collinearity, Maxent also estimates ‘permutation importance’, which uses a jackknife approach to exclude one variable at a time when running the model to assess how much unique information each layer provides. The relative loss of model performance is then scaled and provides another measure of layer importance for a given model.

Model performance can be assessed with a wide variety of statistics that emphasize different aspects of the predictive abilities of a given model [44]. Many model performance statistics, including all of the parameters considered here, are derived from the ‘confusion matrix’, which describes the number of true positives, false positives, true negatives, and false negatives generated by predictions using a certain model [44]. Certain indices of model performance, such as the area under the Receiver Operating Characteristic curve (AUC), do not require a specified threshold to assess the ability of a model to assign a data point (i.e., occurrence data) to a binary state (i.e., presence or absence). Other parameters, such as Kappa (κ; [45]), require a threshold to quantify the performance of binary categorization.

In this study, we evaluated species distribution models using six different indices of model performance that describe different aspects of the ‘confusion matrix’, including both threshold-independent and threshold-dependent variables. These parameters include (1) the threshold-independent area under the Receiver Operating Characteristic curve (AUC) [46]; (2) the area under the curve of the Kappa (κ) at different binary thresholds [45]; (3) the ‘overall performance’ index *sensu* Anderson et al. [47], also known as the correct classification rate [44] at different binary thresholds; (4) the intrinsic commission (false positive) rate [44] at different binary thresholds; (5) the intrinsic omission (false negative) rate [44] at different binary thresholds; (6) and the Pearson’s correlation coefficient between observations in the presence and pseudoabsence data set and the model predictions [48]. Although certain modeling evaluation parameters, such as AUC, have been criticized [49], the variety of parameters considered here allow us to comprehensively evaluate different aspects of model performance, such as model commission (overprediction) and omission (underprediction). Since our research question is focused on determining why Acorn Woodpecker distribution is limited in South America, we placed special emphasis on examining patterns of commission (i.e., overprediction) among our models.

We visualized species distribution models with continuous suitability output from Maxent and also converted continuous suitability into a binary presence-absence model by using the probability threshold that corresponded to maximum Kappa values for each model. Freeman and Moisen [50] found that this method of binary classification performed favorably compared to 10 other methods, such as using the traditional arbitrary cutoff of 0.5 or the threshold at which sensitivity is equal to specificity, based on measurements of predicted prevalence, prediction accuracy, and the resulting distribution output.

## Results

**Fig 2:**
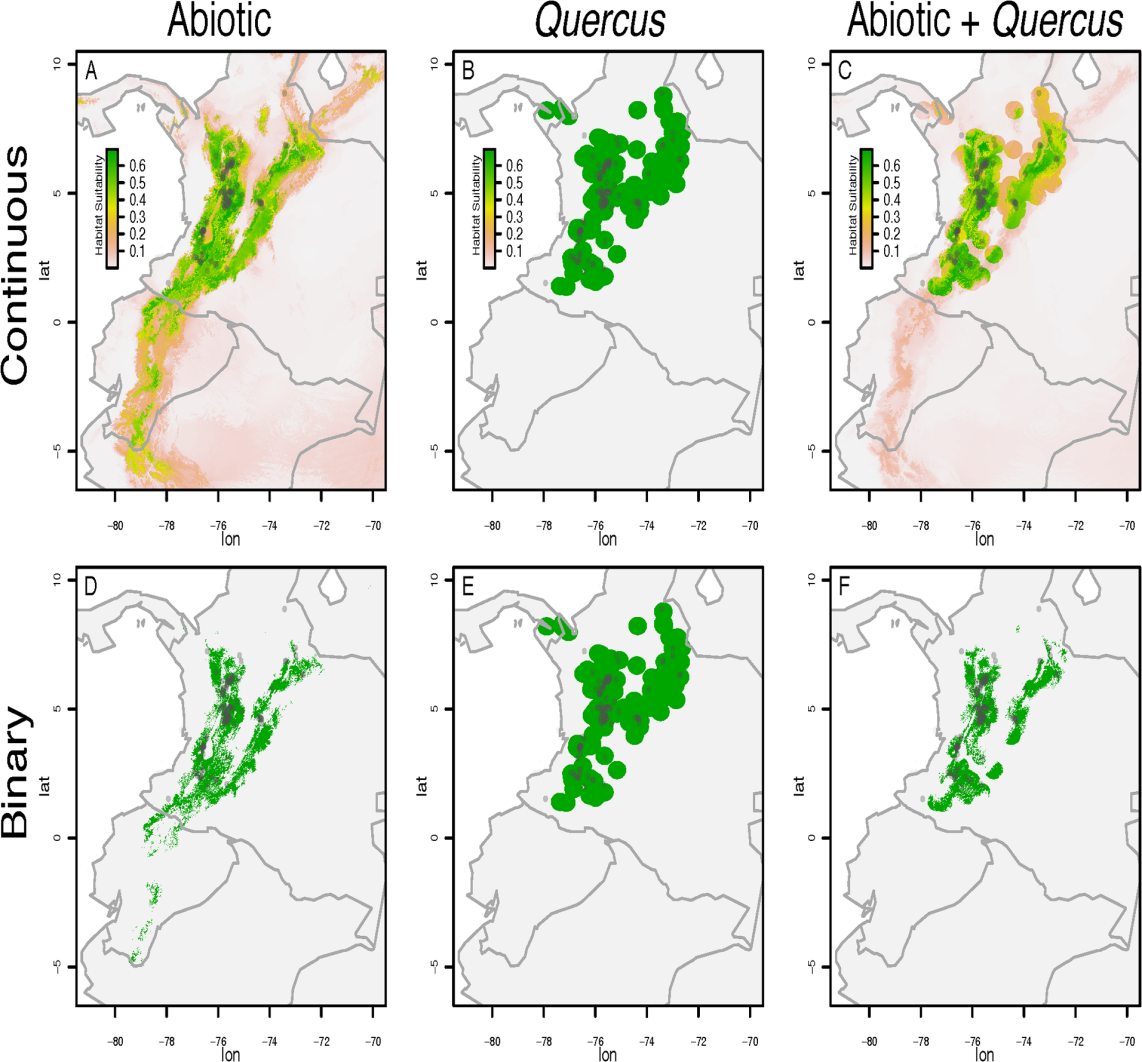
Species distribution models generated for Acorn Woodpecker in northern Andes. Habitat suitability is shown as a continuous variable, in which green colors indicate highly suitable habitat, with (A) abiotic variables alone, (B) *Quercus* presence alone, and (C) abiotic variables + *Quercus* presence. We converted continuous measures of habitat suitability into binary presence and absence models by setting the threshold to the value that maximizes the parameter Kappa, which is an index of model performance. Binary conversions were done for (C) abiotic variables alone and (D) abiotic variables in addition to presence of *Quercus*. Excluding *Quercus* occurrence points generated species distribution models that over-predict the occurrence of Acorn Woodpeckers in South America.

We constructed continuous and binary predictions of habitat suitability for Acorn Woodpeckers in the northern Andes (Fig 2). Response curves indicate how variation among different abiotic and biotic conditions influences the probability of occurrence for Acorn Woodpeckers (S1 Fig).

Using abiotic variables alone, our species distribution model predicted suitable habitat for Acorn Woodpeckers in many regions of Colombia, restricted areas of Venezuela, and high elevation areas in Ecuador and northern Peru (Fig 2A and 2C). The variable that contributed most to the presence-absence predictions of the “abiotic only” model was the mean temperature of warmest quarter, although this variable did not rank highly in permutation importance (Table 1).

**Table 1:** Relative contributions of variables in “abiotic only” species distribution model for Acorn Woodpeckers (*Melanerpes formicivorus*).

Variable	Percent contribution	Permutation importance
BioClim 10: Mean temperature of warmest quarter	56	5.7
BioClim 8: Mean temperature of wettest quarter	15.3	8.9
BioClim 4: Temperature seasonality (standard deviation *100)	15.2	9.5
BioClim 17: Precipitation of driest quarter	5.1	14.6
BioClim 19: Precipitation of coldest quarter	2.4	15.1
BioClim 16: Precipitation of wettest quarter	0.8	14.3
BioClim 14: Precipitation of driest month	0.4	15.4

This model does not include the presence of the Colombian oak (*Quercus humboldtii*). Variables with values > 7.5% for either percent contribution to the species distribution model or permutation importance are included.

In contrast, including Colombian oak presence in addition to abiotic variables largely limited the predicted distribution of Acorn Woodpeckers to the northern Andes in Colombia (Fig 2B and 2D). In this model, the presence of Colombian oak contributed the most to the presence-absence predictions of the model, which was consistent when considering permutation importance (Table 2).

**Table 2:** Relative contributions of abiotic and biotic variables in the “abiotic and Quercus” species distribution model for Acorn Woodpeckers (*Melanerpes formicivorus*).

Variable	Percent contribution	Permutation importance
Colombian oak (*Quercus humboldtii*) presence	79.6	66.3
BioClim 10: Mean temperature of warmest quarter	10.5	4.0
BioClim 3: Isothermality	1.6	8.1
BioClim 17: Precipitation of driest quarter	0.1	8.7

This model includes the presence of Colombian oak (*Quercus humboldtii*) in addition to the 19 abiotic variables from the WorldClim data set. Variables with values > 5% for either percent contribution to the species distribution model or permutation importance are included.

**Fig 3:**
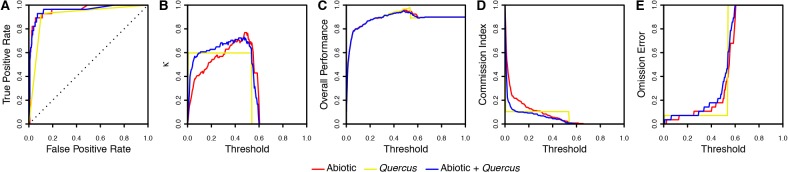
Performance indices of species distribution models constructed from only abotic climatic variables (red), only *Quercus* occurrence data (yellow), and abiotic climatic variables plus *Quercus* occurrence data (blue). Species distribution models that include *Quercus* occurrence data consistently perform better than models constructed from abiotic variables alone based on (A) AUC scores; (B) area under the curve of Kappa values; (C) overall performance *sensu* Anderson et al. [47]; (D) commission (false positive) indices; and (E) omission (false negative) indices. Note that the Pearson’s correlation coefficient is not included in this figure because it does not lend itself to visualization.

We found that including Colombian oak presence as an additional variable generally improved species distribution model performance over species distribution models built from abiotic variables alone (Table 3 and Fig 3). More specifically, while the abiotic-only model had a strong overall performance score, species distribution models that included Colombian oak occurrence points had slightly higher AUC scores, higher Kappa scores, and Pearson’s correlation coefficients (Table 3). Thus, including Colombian oak presences into species distribution modeling improved the ability of models to accurately predict Acorn Woodpecker distribution limits, including its well-documented absence from Ecuador and Peru.

**Table 3:** Performance indices of species distribution models of Acorn Woodpeckers.

Parameter	Abiotic	*Quercus*	Abiotic + *Quercus*
AUC of ROC	0.960	0.912	**0.961**
AUC of Kappa	0.323	0.320	**0.355**
AUC of Overall Performance	**0.879**	**0.879**	0.877
AUC of Commission Index	0.079	0.058	**0.050**
AUC of Omission Index	**0.497**	0.501	0.519
Pearson’s Correlation Coefficient	0.685	0.636	**0.730**

The three species distribution models we compared were constructed from abiotic data alone, *Quercus* occurrence points alone, and abiotic data in combination with *Quercus* occurrence points. Higher scores indicate increased model performance for AUC of ROC, AUC of Kappa, AUC of Overall Performance and Pearson’s correlation coefficient. Lower scores indicate increased model performance for AUC of Commission Index and AUC of Omission index. For each parameter, the best performing models are highlighted in bold. Thus, species distribution models that include *Quercus* occurrence points perform consistently better across a variety of performance indices, particularly with respect to the AUC of Commission Index, which is important for evaluating which factors contribute to the southern distribution limits of Acorn Woodpecker.

## Discussion

We used species distribution models to test the hypothesis that Acorn Woodpecker distribution at its southern range margin is limited by a biotic interaction with Colombian oaks. We found that species distribution models including presence of Colombian oaks as a binary predictor in addition to climatic data had higher AUC, higher kappa, and lower commission rate in northern Ecuador compared to species distribution models built using solely climatic data (Figs 2 and 3), and that the presence of Colombian oaks contributed the most to presence-absence predictions of the niche model built using both climatic data and Colombian oak occurrences (Table 2). These results suggest the Eltonian noise hypothesis may not apply to Acorn Woodpeckers at their southern range margin. Instead, these results are consistent with the hypothesis that the presence of Colombian oaks limits the Acorn Woodpecker at its southern range margin, presumably because the seeds of oaks are an important food resource for Acorn Woodpeckers, as they are in North America [27]. The niche model including Colombian oak presence correctly predicted the presence of Acorn Woodpeckers from regions in the Colombian Andes where Colombian oaks occur and the absence of Acorn Woodpeckers from nearby regions with similar climates that lack oaks (e.g., northern Ecuador).

Additional factors may also influence the location of the Acorn Woodpecker’s southern range margin. For example, dispersal constraints and interspecific competition are two factors that commonly limit distributions of tropical montane birds [51–53]. However, both processes are unlikely to apply to the Acorn Woodpecker. Acorn Woodpeckers are strong dispersers within their North American range [54], though dispersal has not been measured for tropical populations of Acorn Woodpeckers. Moreover, there are no obvious biogeographic barriers to range expansion at its current range margins (i.e., montane forest habitat is continuous along Andean slopes stretching from Colombia south into Ecuador), and nearly all co-occurring montane birds occur in both Colombia and Ecuador [29]. Thus, it is unlikely that the woodpecker’s current range margins in South America reflect dispersal constraints.

A second possibility is that interspecific competition with a closely related species may limit tropical montane birds to smaller distributions despite the availability of suitable environmental space. We do not have rigorous data to address this possibility for Acorn Woodpeckers, but suggest that interspecific competition is unlikely to be a dominant factor influencing the woodpecker’s distribution. Interspecific competition is hypothesized to influence species’ distributions when species are “replaced” geographically by ecologically similar taxa [11,55]. This scenario may apply to the only montane bird species that shares a similar distributional pattern to the Acorn Woodpecker, the Golden-fronted Redstart (*Myioborus ornatus*), which is replaced south of its southern range limit in southern Colombia by its allospecies, the morphologically and ecologically similar Spectacled Redstart (*Myioborus melanocephalus*; [29]). However, Acorn Woodpeckers are the only species of *Melanerpes* woodpecker found in the Andes, and there are no ecologically similar woodpeckers south of the Acorn Woodpeckers’ southern range margin [56]. Thus, it appears unlikely that interspecific competition influences Acorn Woodpecker southern range limit in the northern Andes.

We used commission rates to test the hypothesis that species distribution models using only climatic data overpedict Acorn Woodpecker distribution in the Northern Andes. This analysis hinges on the assumption that our Acorn Woodpecker locality dataset accurately maps Acorn Woodpecker distribution. In general, bird distributions in the Northern Andes are well known thanks both to the efforts of field ornithologists and legions of birdwatchers [29]. This is particularly true in northern Ecuador, which is a popular location for birdwatching [56]. Within birds, Acorn Woodpeckers are a particularly conspicuous species due to their social behavior and loud vocalizations, and are thus easily detectable when present. It is therefore likely that sites outside the known range of Acorn Woodpeckers but predicted as suitable for Acorn Woodpeckers in our models (especially in northern Ecuador) represent model overpredictions rather than sites where Acorn Woodpeckers occur but have yet to be detected. This assumption could be further tested by directed field surveys for Acorn Woodpeckers in the Andes of extreme southern Colombia and northern Ecuador.

## Conclusions

Our results support the hypothesis that a biotic interaction with an important food resource limits a tropical bird’s large-scale geographic distribution. Tropical species have been hypothesized to inhabit distributions largely shaped by biotic interactions [57]. However, few studies have marshaled quantitative evidence that biotic interactions limit distributions of tropical species [58–60], and the influence of biotic interactions with important food resources on the distributional limits of tropical birds has seldom been previously considered in a species distribution modeling framework (but see [24]). It is especially intriguing that Acorn Woodpeckers in the Northern Andes are apparently limited by the presence of Colombian oaks despite their generalist diet—tropical populations of Acorn Woodpeckers flycatch for insects, drink sap, consume acorns, fruit, grains and eat an array of insects [30,61]. Thus, although Acorn Woodpeckers in Colombia can occur at sites where oaks are absent [30], and tropical populations of Acorn Woodpeckers do not store acorns in large granary trees as do North American populations [30,62], oaks appear to be a sufficiently important food resource that they influence Acorn Woodpecker distribution at a broad geographic scale, perhaps because Acorn Woodpeckers regularly drink sap from Colombian oak trees [30]. Further studies should investigate Acorn Woodpecker diet in the Northern Andes. Regardless, our results suggest that a biotic interaction with oaks limits Acorn Woodpecker broad geographic distribution but may not influence local patterns of abundance and distribution within the northern Andes, a reversal of the commonly noted pattern that biotic interactions influence local patterns of abundance and distribution but not broad geographic patterns [3].

We conclude with a call for further case studies to test whether biotic interactions influence large-scale distributional limits (e.g., [22–25]). Such studies are especially appropriate in birds, where reciprocal transplant experiments to experimentally assess the impact of biotic interactions on distributional limits are nearly impossible, but voluminous distributional data facilitates construction of species distribution models to explore how biotic interactions impact species’ distributional limits. Acorn Woodpeckers may be extreme in their apparent reliance on a particular species of tree as a food resource. However, we suggest that many tropical birds may inhabit distributions that are limited more by the presence of single plant species that are important food resources than by abiotic climatic factors. For example, a number of tropical bird species are specialized on bamboo seeds (Neotropics; [63]: Asian tropics;[64]: Melanesia; [65]; these birds likely inhabit distributions limited more by bamboo distribution than by climatic factors [66]. Similar scenarios may apply to other specialist foragers, such as *Phaethornis* hermits (hummingbirds) that forage primarily on *Heliconia* flowers [67], or the Giant Conebill (*Oreomanes fraseri*) and Point-tailed Palmcreeper (*Berlepschia rikeri*) that are tightly associated with, respectively, high-elevation *Polylepis* forests in the Andes and *Mauritia* palm groves in the Amazon [29]. Future research will determine the extent to which biotic interactions with plants that provide important food sources structure distributions of tropical montane birds.

## Supporting Information

S1 Fig Response curves for predictor variables (BioClim variables and Colombian oak presence) in species distribution models. Red lines correspond to the abiotic-only models, yellow lines correspond to the *Quercus-*only model, and blue lines correspond to the abiotic + *Quercus* model.
(TIFF)

S1 File Occurrence data of Acorn Woodpeckers (*Melanerpes formicivorus*) used in this study. This database includes records from both museum specimens and eBird records. Specimen records have unique Global Biodiversity Information Facility (GBIF) identification numbers (“GBIF_ID”); eBird records also have unique identification numbers (“eBird_ID”). There are three types of eBird records—Stationary Counts, Traveling Counts and Exhaustive Area Counts.
(XLSX)

S2 File Occurrence data of Colombian Oak (*Quercus humboldtii*) used in this study. This database was developed and published by Gonzalez et al. (35). In addition to latitude and longitudes for each record, Gonzalez et al (35) provided information on the collector, herbarium and identification number of each record (when available).
(XLSX)
