# Supplementary figures and images for: The Geographic Distribution of a Tropical Montane Bird Is Limited by a Tree: Acorn Woodpeckers (Melanerpes formicivorus) and Colombian Oaks (Quercus humboldtii) in the Northern Andes
Source: PLoS One. 2015 Jun 17;10(6):e0128675. doi: 10.1371/journal.pone.0128675 (PMC4471272; doi:10.1371/journal.pone.0128675)

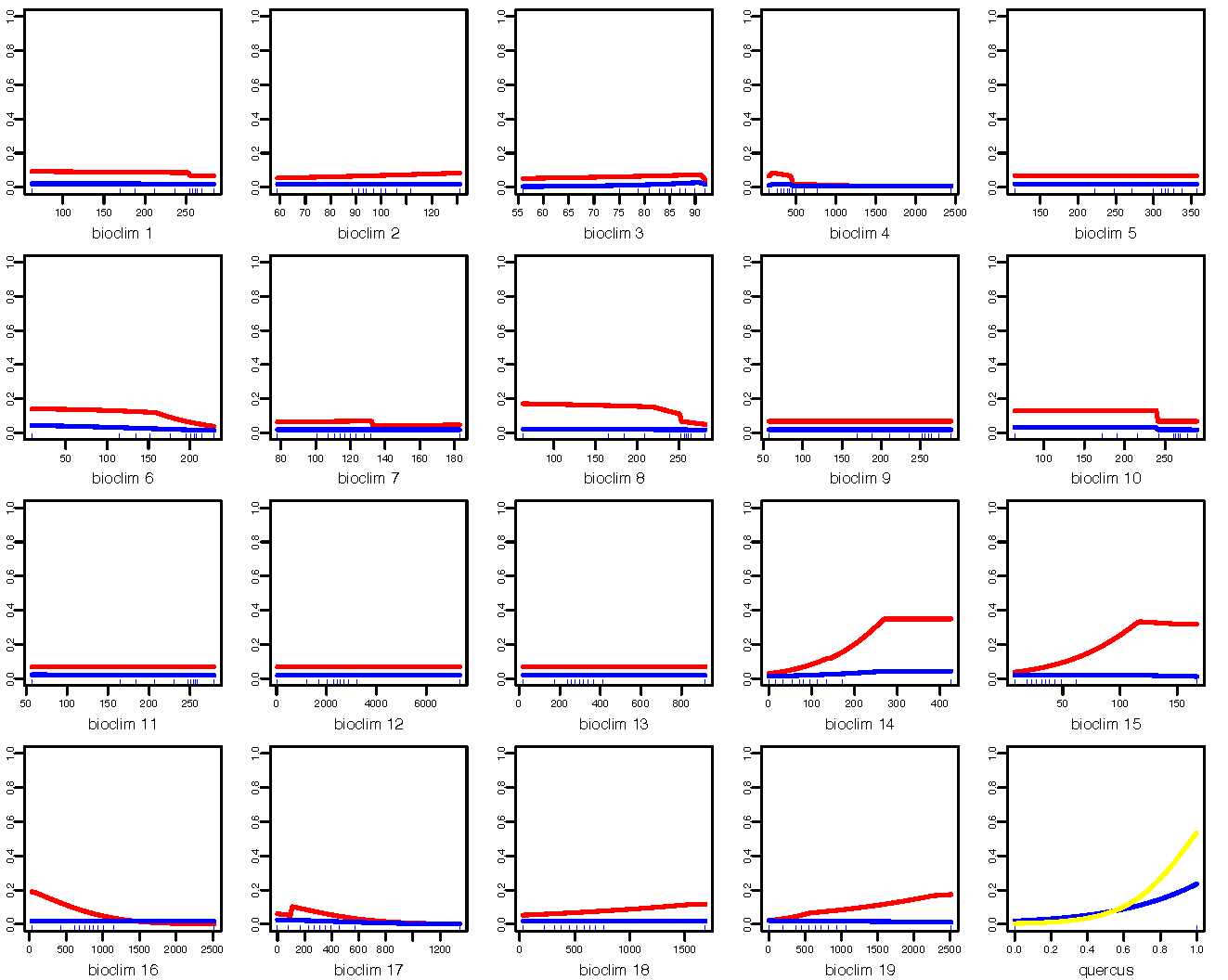

Supplement: S1 Fig — Red lines correspond to the abiotic-only models, yellow lines correspond to the Quercus-only model, and blue lines correspond to the abiotic + Quercus model. (TIFF) [file pone.0128675.s001.tiff]
